# Complete chloroplast genomes of the *Chlamydomonas reinhardtii* nonphotosynthetic mutants CC-1375, CC-373, CC-4199, CC-2359 and CC-1051

**DOI:** 10.1080/23802359.2017.1347838

**Published:** 2017-07-11

**Authors:** Alexandra E. DeShaw, Francisco Figueroa-Martinez, Adrian Reyes-Prieto

**Affiliations:** a Department of Biology, University of New Brunswick, Fredericton, Canada;; b CONACyT Research Fellow?Universidad Autonoma Metropolitana, Mexico City, Mexico;; c Integrated Microbiology Program, Canadian Institute for Advanced Research, Toronto, Canada

**Keywords:** Nonphotosynthetic algae, *Chlamydomonas*, colorless mutants, genome erosion, genome reduction

## Abstract

The chloroplast genomes (cpDNA) of five *Chlamydomonas reinhardtii* nonphotosynthetic mutants were sequenced. The architecture, gene content, and synteny of the cpDNAs from the five mutants are identical to the *C. reinhardtii* ‘wild-type’ plastome. A small number of differences at sequence level between coding regions of the reference genome and the cpDNAs of the mutants were detected. The vast majority of the sequence differences were synonymous and likely due to nucleotide substitutions preceding the generation of the mutant strains, but not caused by the erosion of the cpDNA following the loss of photosynthesis.

*Chlamydomonas* nonphotosynthetic mutants are suitable experimental models for studying the evolution of colourless algae (Figueroa-Martinez et al. 2015). To gain insights into the erosion and reduction processes of chloroplast genomes (cpDNA) following the loss of photosynthesis, we sequenced the cpDNAs of five *C. reinhardtii* mutants with nonphotosynthetic phenotypes. The group of analyzed strains included two plastome mutants, CC-1375 ac-u-lambda (*psb*A) and mt + CC-373 ac-u-c-2-21 mt + (atpB), and three variants with alterations in nuclear loci, CC-1051 M18 (locus ac9) mt+, CC-2359, lts1-30 mt- (locus PSY1), and CC-4199 lts1-204 (PSY1) (Figure 1). Mutant strains were acquired from the *Chlamydomonas Resource Center* (www.chlamycollection.org).

**Figure 1.**
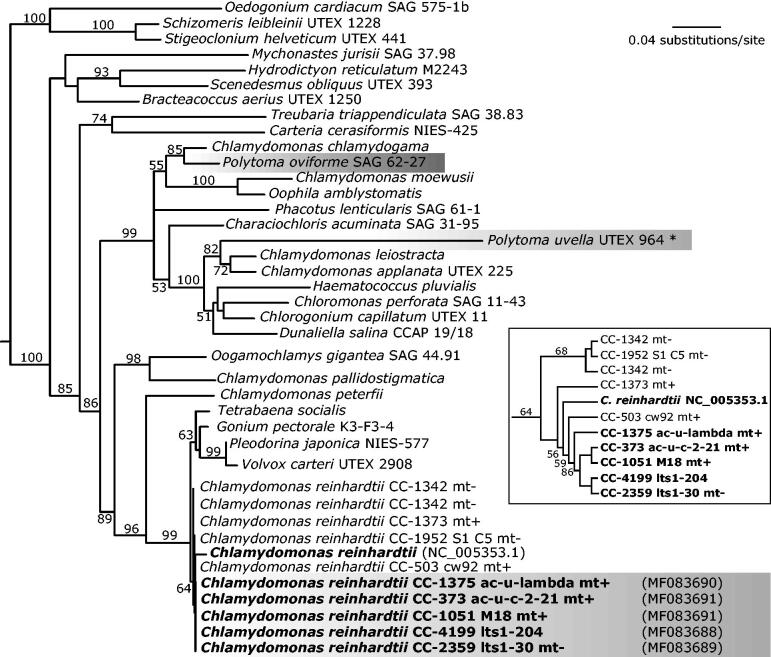
Maximum likelihood tree of diverse algae of the order Chlamydomonadales estimated using sequences of the chloroplast 16S ribosomal RNA (*rrs* gene). Nucleotide sequences were aligned with MAFFT v7 (Katoh and Standley 2013) and manually refined. Maximum likelihood (ML) tree was estimated with the RAxML v8.2 (Stamatakis 2014) considering the GTR + G substitution model. Branch support was assessed with 500 bootstrap replicates. Numbers near nodes indicate ML bootstrap support (only values >50% are shown). Branch lengths are proportional to the number of substitutions per site indicated by the scale bar. Gray boxes highlight nonphotosynthetic species and strains. The inset tree shows a cladogram detailing the *Chlamydomonas reinhardtii* lineage, which includes the five nonphotosyntehitc mutants (bold font) analyzed in in the present work. The DDBJ/EMBL/GenBank accession numbers of the complete chloroplast genomes are in parenthesis.

Total DNA from each strain was purified using phenol-chloroform extraction methods (Figueroa-Martinez et al. 2017). Paired-end libraries were prepared and sequenced at Genome Quebec (McGill University, Montreal, Quebec) using Illumina technology (Hiseq2500; Illumina; San Diego, CA). Sequence quality (∼60.6 × 10^6^ reads per strain) was evaluated with FastQC v0.11.5 (Andrews 2016). High-quality reads (Phred score 28, length >70 bp) were assembled with Ray v2.2.0 (Boisvert et al. 2010) using 21 and 31 kmers. Gap bridging by read coverage was performed with Geneious v8 (Kearse et al. 2012). Coding regions were annotated using the 203.8 kb sequence of the ‘wild-type’ *C. reinhardtii* cpDNA (Maul et al. 2002) as reference; tRNA’s were predicted using tRNAscan-SE v1.21 (Lowe and Eddy 1997) and rRNA’s using RNAweasel (Centre Robert-Cedergren Bio-informatique et Génomique, Université de Montréal 2014). The new cpDNA sequences are deposited in DDBJ/EMBL/GenBank under accession numbers MF083688 to MF083692.

The five cpDNAs of the mutants present the same quadripartite structure of the reference genome. Gene content (66 unique protein-coding genes, 5 rRNA, 27 tRNAs), introns, and synteny are conserved. Besides the expected reduced length of the CC-373 and CC-1375 cpDNAs, the plastomes from the other three mutants are also slightly smaller (<199 kb) than the reference. If we exclude the mutant loci, all 64 plastid protein-coding genes are identical among the mutants. In contrast, 17 coding regions from the reference cpDNA presented at least one (at most nine) nucleotide substitution with respect to the mutant sequences. Most detected substitutions were synonymous and therefore likely fixed independently in the different strains used to assemble the reference cpDNA (Maul et al. 2002), which are not the same used to produce the five nonphotosynthetic mutants (Chemerilova 1978; Shepherd et al. 1979; Girard et al. 1980; Myers et al. 1982; McCarthy et al. 2004).

Deletions of the plastid loci *atp*B (CC-373) and *psb*A (CC-1375) were corroborated. Coincidently, CC-1375 presents a point deletion in *atp*B that causes a frame shift and a premature stop codon. It was not possible to discern if this mutation arose as consequence of genome erosion following the loss of photosynthesis, or if it was produced by the same mutagenic method that caused the *psb*A deletion. No additional mutations were identified in the cpDNAs of the three nuclear mutants. The lack of evident erosion in the cpDNAs of the mutants is likely due to the relatively low number of generations that have passed since the loss of photosynthesis and the mild selective conditions under which the cultures are maintained.
